# Efficacy of Tumor-Targeting *Salmonella typhimurium* A1-R against Malignancies in Patient-Derived Orthotopic Xenograft (PDOX) Murine Models

**DOI:** 10.3390/cells8060599

**Published:** 2019-06-16

**Authors:** Takashi Murakami, Yukihiko Hiroshima, Kentaro Miyake, Tasuku Kiyuna, Itaru Endo, Ming Zhao, Robert M. Hoffman

**Affiliations:** 1 AntiCancer, Inc., San Diego, CA 92111, USA; impressor@hotmail.co.jp (T.M.); yhiroshiy13@gmail.com (Y.H.); miyekentarou@gmail.com (K.M.); ultimum50@gmail.com (T.K.); mingz@hotmail.com (M.Z.); 2 Department of Gastroenterological Surgery, Graduate School of Medicine, Yokohama City University, Yokohama 236-0004, Japan; endoit@yokohama-cu.ac.jp; 3 Department of Surgery, University of California, San Diego, CA 92093, USA

**Keywords:** *Salmonella typhimurium* A1-R, tumor-targeting, patient-derived orthotopic xenograft, malignancy, bacterial therapy

## Abstract

We developed tumor-targeting *Salmonella typhimurium* (*S. typhimurium*) A1-R, a facultative anaerobe that is an auxotroph of leucine and arginine. The tumor-targeting efficacy of *S. typhimurium* A1-R was demonstrated in vivo and vitro using several malignant cell lines including melanoma, sarcoma, glioma, breast, pancreatic, colon, cervical, prostate, and ovarian cancers. Our laboratory also developed a patient-derived orthotopic xenograft (PDOX) model by implanting patient-derived malignant tumor fragments into orthotopic sites in mice. We reviewed studies of *S. typhimurium* A1-R against recalcitrant cancers. *S. typhimurium* A1-R was effective against all PDOX tumor models tested and showed stronger efficacies than chemotherapy or molecular-targeting therapy against some tumors. Furthermore, the synergistic efficacy of *S. typhimurium* A1-R when combined with chemotherapeutic agents, molecular-targeting agents, or recombinant methioninase was also demonstrated. We suggest potential clinical uses of this *S. typhimurium* A1-R treatment.

## 1. Introduction

Dr. William B. Coley began bacterial therapy of cancer using *Streptococcus pyogenes* (*S. pyogenes*). He then developed Coley’s toxin, a mixture of killed *S. pyogenes* and *Serratia marcescens*, achieving clinical responses for many malignant tumors [1].

Live bacteria can actively penetrate tumors to reach lesions distant from blood vessels, where chemotherapeutic drugs cannot be delivered, and damage malignant cells by several cytotoxic mechanisms [2]. Obligate anaerobes and facultative anaerobes can have intrinsic tumor-targeting ability because they can survive intratumor hypoxia. By contrast, traditional treatments such as chemotherapy or radiation therapy have reduced efficacy in hypoxic regions in tumors where malignant cells are generally quiescent. Moreover, the immune-suppressive tumor microenvironment is conducive to bacteria, and host immune responses against administered bacteria may enhance antitumor immunity [3,4,5]. These specific advantages in bacterial therapy can overcome the limit of traditional treatments, even exerting synergistic efficacy in combination with these treatments.

In clinical studies, limited antitumor efficacy has been shown thus far. *Clostridium. butyricum* (*C. butyricum*) M-55 resulted in oncolysis and accumulation in treated tumors [6,7]. Intravenous or intratumoral administration of *C. novyi*-NT induced intratumor infection and necrosis [7]. Moreover, *Salmonella typhimurium* (*S. typhimurium*) VNP20009 attenuated by *msbB* and *purI* mutations, showed bacterial colonization of treated melanomas [8,9]. Objective tumor response was not observed in any of these studies, however, several studies using *S. typhimurium* showed bacterial colonization in treated tumors after local or systemic administration [7].

*S. typhimurium* is a facultative anaerobe. Green fluorescence protein (GFP)-labeled *S. typhimurium* A1-R developed by our laboratory has high tumor-targeting efficacy, due to the leucine–arginine auxotroph, resulting in broad antitumor efficacy and limited adverse effects [10]. In a CT26 colon cancer-bearing BALB/c mouse experiment, *S. typhimurium* A1-R was quickly eliminated from normal organs including the liver and spleen seven days after intravenous administration [11]. In contrast, *S. typhimurium* A1-R remained at high density in CT26 tumors. In addition, tumors treated with *S. typhimurium* A1-R were significantly smaller than those treated with *S. typhimurium* VNP20009. The efficacy of *S. typhimurium* A1-R was demonstrated in orthotopic nude mouse models of prostate [12], breast [13,14], pancreatic [15,16], and ovarian cancer [17], as well as in sarcomas [18] and gliomas [19,20]. *S. typhimurium* A1-R was also effective in metastatic cancer models [21,22].

We review in the present report the therapeutic efficacy of *S. typhimurium* A1-R against malignancies in patient-derived orthotopic xenograft (PDOX) nude mouse models, in which human tumors are orthotopically implanted in mice, to examine future clinical applicability.

## 2. *S. typhimurium* A1-R against PDOX Tumor Models

### 2.1. Overview

We established PDOX tumors as follows: when the original tumors derived from primary sites, the PDOX tumors were implanted into the same primary sites in mice; when the original tumors derived from recurrent or metastatic sites, the PDOX tumors were implanted into original, recurrent, or metastatic sites in mice.

From 2014 to 2018, a total of 17 articles describing the efficacy of *S. typhimurium* A1-R in PDOX models were identified. All of the 17 studies of human cancer of different histological types were evaluated and the efficacy of *S. typhimurium* A1-R was compared with the efficacy of chemotherapy (Table 1 and Table 2) [23,24,25,26,27,28,29,30,31,32,33,34,35,36,37,38,39]. There were six soft tissue sarcoma (STS) PDOX studies, including rare type tumors [25,27,28,33,37,39], four melanoma PDOX studies [26,29,30,34], three pancreatic cancer PDOX studies [23,24,36], two osteosarcoma PDOX studies [31,35], a gastrointestinal stromal tumor (GIST) PDOX study [32], and a carcinoma of unknown primary (CUP) PDOX study [38]. Ten of the 17 tumors were derived from primary sites [23,24,26,27,28,29,30,34,36,37], while the remaining seven tumors were recurrent or metastatic [25,31,32,33,35,38,39]. Two PDOX models from different cancer types were established from the tumors grown in distant lesions [31,35]. Some tumors were identified to have specific genetic alterations [26,29,30,32,33].

### 2.2. Establishment of PDOX Models

Fresh patient-derived tumor tissue samples were immediately transported to the lab on ice. The samples were cut into 5 mm fragments and implanted subcutaneously into nude mice to establish. They were then passaged when they grew to approximately 10 mm. The grown tumors were cut into small fragments and implanted orthotopically into nude mice. 

**Figure 1:**
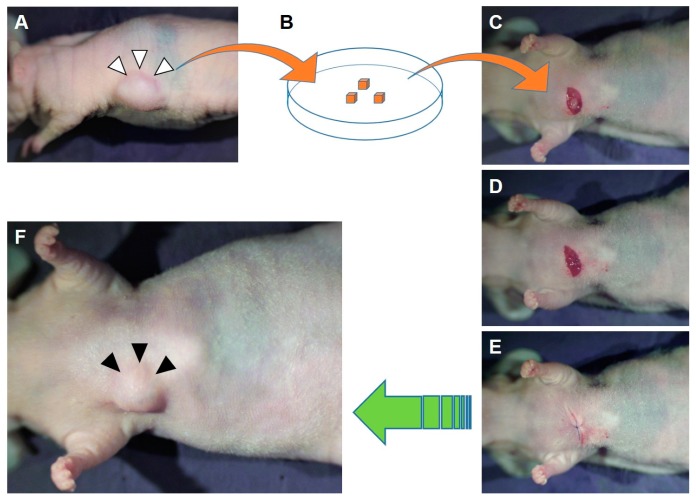
Procedures to establish a Ewing’s sarcoma patient-derived orthotopic xenograft (PDOX) model. A subcutaneously grown patient-derived Ewing’s sarcoma was resected (**A**) and cut into small fragments on a dish (**B**). A 7 mm skin incision was made on the right chest wall and then a single tumor fragment was implanted orthotopically into the layer between the pectoral and intercostal muscles in the right chest wall of nude mouse (**C**). After the pectoral muscle was closed by 6–0 nylon sutures (**D**), the skin incision was sutured (**E**). (**F**) The established PDOX model 4 weeks after the orthotopic implantation [28]. White arrowheads indicate the subcutaneously grown tumor. Black arrowheads indicate the established PDOX tumor.

For example, procedures for the establishment of a Ewing’s sarcoma PDOX model are shown in Figure 1 [28]. The established subcutaneous tumor was cut into small fragments (Figure 1A,B). A seven mm skin incision was made on the right chest wall; then, a single tumor fragment was implanted orthotopically into the layer between the pectoral and intercostal muscles in the right chest wall of the nude mouse to establish a PDOX model (Figure 1C–E). The implanted tumor was grown in the right chest wall in the PDOX model (Figure 1F).

**Figure 2:**
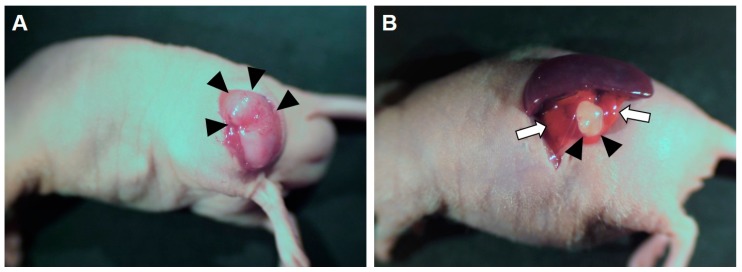
Examples of PDOX models. (**A**) A soft tissue sarcoma PDOX model was established by implanting a tumor into the lower extremity of a nude mouse [27]. The tumor was exposed by cutting the skin. (**B**) A pancreatic cancer PDOX model was established by implanting a tumor into the tail of the pancreas of a red fluorescent protein (RFP)-expressing nude mouse [24]. Black arrowheads indicate grown tumors. White arrows indicate the RFP-expressing pancreas.

For STSs, tumor tissue was implanted into the upper or lower extremities in five experiments (Figure 2A) while the metastatic Ewing’s sarcoma shown above was implanted into the chest wall of mice [25,27,28,33,37]. A metastatic melanoma was also implanted into the chest wall of mice [26,29,30,34]. Pancreatic cancers were implanted into the tail of the pancreas of mice (Figure 2B) [23,24,36]. Osteosarcoma lung metastases originating from a primary lesion in the femur were implanted both in the femur and the lung [31,35]. A GIST tumor derived from recurrent lymph nodes and originating from a primary gastric tumor was implanted orthotopically into the gastric wall [32]. A PDOX of a cancer of unknown primary origin was established by implanting tumor fragments into the neck lymph node of mice, which was the metastatic site of the patient [38].

### 2.3. S. typhimurium A1-R Therapy

*S. typhimurium* A1-R was administered via an intravenous (i.v.) [23,26,28,29,30,31,32,33,34,35,36,37,38,39], intraperitoneal (i.p.) [24,25], intratumoral (i.t.) [27,28], or intra-arterial (i.a.) injection [31]. A single dose of *S. typhimurium* A1-R ranged from 5 × 10^5^ colony-forming units (CFUs) to 1.5 × 10^8^ CFUs. Intravenous injection twice weekly with a dose of 5 × 10^7^ CFUs was used in most experiments. *S. typhimurium* A1-R treatment was performed either as a monotherapy or a polytherapy in combination with chemotherapeutic or molecular-targeting agents, or recombinant methioninase (rMETase), which reduces the plasma methionine on which cancer cells are addicted [40]. Polytherapy was performed in a concurrent or metachronous manner. 

### 2.4. S. typhimurium A1-R Treatment Efficacies

#### 2.4.1. Tumor-Targeting Efficacy

**Figure 3:**
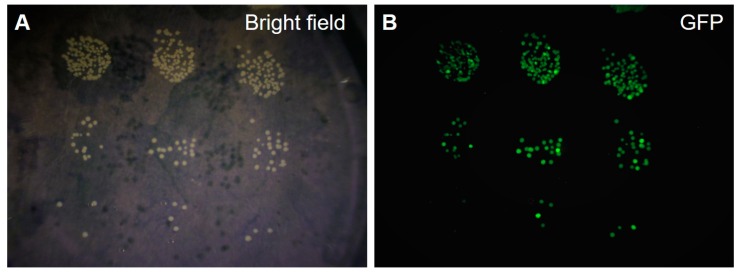
Agar culture from a tumor treated with S. typhimurium A1-R in a Ewing’s sarcoma PDOX model. Bright field (**A**) and fluorescence imaging (**B**) of cultured green fluorescent protein (GFP)-expressing S. typhimurium A1-R targeted to a Ewing’s sarcoma PDOX tumor [28].

The tumor-targeting efficacy of *S. typhimurium* A1-R was evaluated via culture of resected specimens. A fluorescent microscope was used to detect GFP-expressing *S. typhimurium* A1-R in tumors grown in PDOX models treated with *S. typhimurium* A1-R i.t., i.p., i.v., or i.a. injection [25,26,28,29,30,31,32,33,34,37,38]. Figure 3 shows bright field and fluorescent imaging of cultured GFP-expressing *S. typhimurium* A1-R grown from a Ewing’s sarcoma PDOX tumor [28]. Abundant *S. typhimurium* A1-R were present in STS PDOX tumors, including undifferentiated STS [37], Ewing’s sarcoma [28], pleomorphic liposarcoma [33], follicular dendritic-cell sarcoma [25], melanoma PDOX tumors [26,29,30,34], an osteosarcoma PDOX [31], a GIST PDOX [32], and a CUP PDOX [38]. By contrast, GFP-expressing *S. typhimurium* A1-R were not detectable in adjacent muscles, suggesting selective tumor-targeting efficacy [31,37]. 

#### 2.4.2. Antitumor Efficacy of *S. typhimurium* A1-R Compared to Untreated Control

The treatment efficacy of S. typhimurium A1-R was confirmed in all PDOX models (Table 2). Significant tumor growth inhibition occurred after i.v., i.p., i.t., and i.a. injection of S. typhimurium A1-R. Interestingly, i.v. administration tended to be more effective compared to i.t. administration in the Ewing’s sarcoma PDOX models [28]. In addition, S. typhimurium A1-R showed stronger efficacy administered i.a. than i.v. injection in the osteosarcoma lung metastasis PDOX models [31].

#### 2.4.3. Antitumor Efficacy of *S. typhimurium* A1-R Compared to Chemotherapy or Molecular-Targeting Therapy

*S. typhimurium* A1-R showed stronger antitumor efficacy than gemcitabine, cisplatinum, or fluorouracil treatment in the pancreatic PDOX model [23]. In the osteosarcoma PDOX models, *S. typhimurium* A1-R was more effective than cisplatinum [31,32]. In the STS PDOX models, *S. typhimurium* A1-R resulted in greater tumor growth inhibition compared to doxorubicin treatment [33,35,39]. Moreover, *S. typhimurium* A1-R showed stronger efficacy than imatinib in the GIST PDOX model [37].

#### 2.4.4. Synergistic Antitumor Efficacy of *S. typhimurium* A1-R in Combination with Other Agents

Synergistic treatment efficacy was observed with combination of *S. typhimurium* A1-R with chemotherapy [23,36], molecular-targeting agents [23,26,29,30], or rMETase [34,35]. In pancreatic cancer PDOX models, *S. typhimurium* A1-R had additional efficacy when combined with gemcitabine or gemcitabine plus bevacizumab [24,36]. Additionally, the combination treatment of *S. typhimurium* A1-R with temozolomide or vemurafenib significantly reduced tumor growth compared to monotherapy with these agents [26,29,30]. Triple therapy using *S. typhimurium* A1-R, rMETase, and cisplatinum was more effective than double therapy using *S. typhimurium* A1-R with rMETase, or monotherapy of these agents [32].

#### 2.4.5. Histological Effects

**Figure 4:**
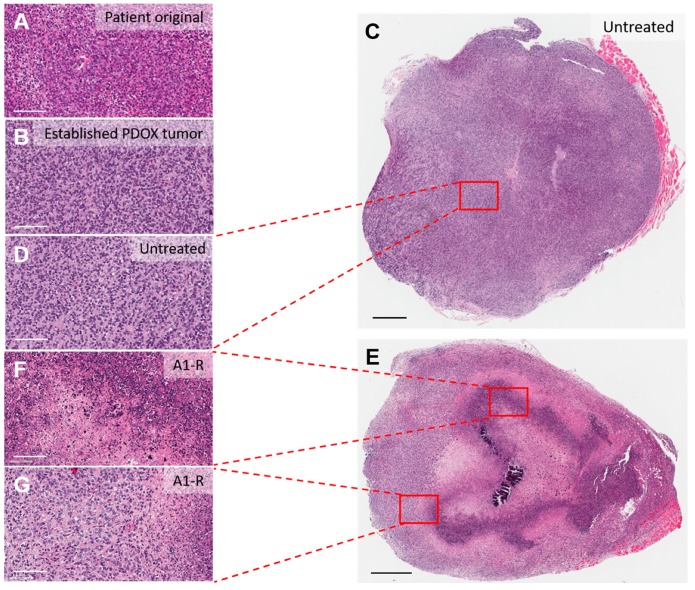
Histological findings in untreated tumors and tumors treated with *S. typhimurium* A1-R in the Ewing’s sarcoma PDOX model (**H and E** staining). (**A**) Ewing’s sarcoma from the original patient tumor. (**B**) High-magnification image of an established tumor in a Ewing’s sarcoma PDOX model. (**C**) Whole tumor image of untreated control tumor in a Ewing’s sarcoma PDOX model. (**D**) High-magnification image of (**C**). (**E**) Whole tumor image of a *S. typhimurium* A1-R-treated tumor in a Ewing’s sarcoma PDOX model. (**F**,**G**) High-magnification images of (E). Scale bars in (A,B,D,F,G): 100 μm; scale bars in (**C**,**E**): 500 μm.

Established tumors in the PDOX model had a similar morphologic appearance to the original patient tumor (Figure 4A,B). *S. typhimurium* A1-R-treated tumors showed extended necrosis compared to untreated tumors. As an example, *S. typhimurium* A1-R caused central tumor necrosis to a large extent in the Ewing’s sarcoma PDOX model, while the untreated tumors grew without necrosis (Figure 4C–G). In a study of an osteosarcoma lung metastasis PDOX model, *S. typhimurium* A1-R treatment resulted in changes in sarcoma cell shape but not necrosis [35]. *S. typhimurium* A1-R treatment in combination with cisplatinum and rMETase resulted in tumor necrosis. Moreover, *S. typhimurium* A1-R showed more extensive necrosis when combined with chemotherapy or molecular-targeting agents than *S. typhimurium* A1-R monotherapy on undifferentiated STS and melanoma PDOX models [27,29,30]. When compared to standard treatment, *S. typhimurium* A1-R induced a higher degree of necrosis in several PDOX models including pancreatic cancer, STS, osteosarcoma, and GIST [24,25,31,32,33,37]. The i.a. administration of *S. typhimurium* A1-R led to more extensive necrosis than intravenous administration in the osteosarcoma PDOX model [31]. These results indicate that tumor necrosis was generally associated with tumor growth suppression.

### 2.5. Adverse Effects Caused by S. typhimurium A1-R Treatment

None of the PDOX experiments showed adverse effects, in terms of significant weight loss, in mice treated with *S. typhimurium* A1-R compared to the untreated control.

## 3. Conclusions and Future Perspectives

PDOX models are theoretically better at mimicking the human disease than heterotopic tumors, increasing the robustness of drug discovery studies. The present review demonstrates the strong antitumor efficacy of *S. typhimurium* A1-R against recalcitrant-caner PDOX models, indicating advantages that *S. typhimurium* A1-R may have over chemotherapy. Therefore, for rare malignancies or cancers of unknown primary origin, for which effective treatments have not been established, *S. typhimurium* A1-R treatment can be a good candidate. Moreover, for highly aggressive malignancies such as pancreatic cancer or melanomas, *S. typhimurium* A1-R was highly effective when combined with chemotherapy or molecular-targeting therapy. In addition, adverse effects were shown to be limited. Therefore, *S. typhimurium* A1-R treatment has clinical potential. 

*S. typhimurium* penetrated the cancer cells in vitro by being attracted to small molecules such as ribose and serine [41,42]. The present review demonstrated how *S. typhimurium* A1-R targeted tumors in several PDOX mouse models. The antitumor efficacy of *S. typhimurium* A1-R against many kinds of cancer cell lines was demonstrated and suggested that *S. typhimurium* A1-R kills cancer cells directly [13,43,44]. Unchugonova et al. demonstrated that cancer cells infected by *S. typhimurium* A1-R expanded and burst, resulting in loss of viability [45]. Importantly, *S. typhimurium* A1-R, a facultative anaerobe, can grow under anaerobic condition [13,44]. As a result, *S. typhimurium* A1-R induces central tumor necrosis (Figure 4). In addition, *Salmonella* plays the role of inducing antitumor immune responses in an immunocompetent model [46]. *Salmonella* enhances both innate and adaptive immunity. *Salmonella* induces cytokine production, including interferon-γ, via Toll-like receptor 4 signaling [5]. Upregulated cytokines contribute to the recruitment of peripheral immune cells to the tumor [5,47]. Avogadri et al. demonstrated that intratumoral injection of *S. typhimurium* resulted in recruitment of CD8^+^ lymphocytes, CD4^+^ lymphocytes and B lymphocytes as well as macrophages and granulocytes in the tumor [3]. We also demonstrated that the antitumor efficacy of *S. typhimurium* A1-R was correlated with CD8^+^ lymphocyte infiltration into treated tumors in a pancreatic cancer syngeneic immunocompetent mouse model [48]. Moreover, *S. typhimurium* A1-R acts as a decoy. It induces the cancer cells to leave the chemo-sensitive state of the cell cycle, making the cancer cells highly sensitive to chemotherapy [49]. These facts suggest that various mechanisms are involved in the antitumor efficacy of *S. typhimurium* A1-R. 

The present review discusses the antitumor efficacy of *S. typhimurium* A1-R against recalcitrant cancer PDOX models. Pre-clinical efficacy studies of *S. typhimurium* A1-R were completed and only a toxicity test needs to be performed to enable *S. typhimurium* A1-R to begin phase I clinical studies.

## Figures and Tables

**Table 1:** Summary of studies in which *S. typhimurium* A1-R was administered to PDOX models.

		Patient			Mouse
Tumor Type (Subtype)	Year	Origin	Original Site	Genetics	Implanted Site
**Pancreatic Cancer**					
	2014 [23]	Primary	Pancreas	VEGF+	Pancreas
	2014 [24]	Primary	Pancreas	-	Pancreas
	2018 [36]	Primary	Pancreas	-	Pancreas
**Soft Tissue Sarcoma**					
(FDCS)	2016 [25]	Recurrent/regional	Lower extremity (Primary site: lower extremity)	-	Lower extremity
(UPS)	2016 [27]	Primary	Lower extremity	-	Lower extremity
(Ewing’s sarcoma)	2017 [28]	Primary	Chest wall	-	Chest wall
(Pleomorphic liposarcoma)	2018 [33]	Recurrent/regional	Upper extremity (Primary site: upper extremity)	PDGFRA amplification	Upper extremity
(USTS)	2018 [37]	Primary	Lower extremity	-	Lower extremity
(Myxofibrosarcoma)	2018 [39]	Recurrent/regional	Upper extremity	-	Upper extremity
**Melanoma**					
	2016 [26]	Primary	Chest wall	BRAF-V600E mutation	Chest wall
	2017 [29]	Primary	Chest wall	BRAF-V600E mutation	Chest wall
	2017 [30]	Primary	Chest wall	BRAF-V600E mutation	Chest wall
	2018 [34]	Primary	Abdominal wall	BRAF-V600E mutation negative	Abdominal wall
**Osteosarcoma**					
	2017 [31]	Recurrent/distant	Lung (Primary site: femur)	-	Femur
	2018 [35]	Recurrent/distant	Lung (Primary site: femur)	-	Lung
**GIST**					
	2018 [32]	Recurrent/regional	Lymph node (Primary site: stomach)	c-kit (exon 11 and 17) mutation	Gastric wall
**Cancer of Unknown Primary**					
	2018 [38]	Metastatic	Neck lymph node (Primary site: unknown)	-	Left supraclavicular fossa

**Table 2:** **Efficacy of***S. typhimurium* A1-R was administered on PDOX models.

	*S. typhimurium* A1-R			
Tumor Type (Subtype)	Route	Dose	Mono- or Polytherapy	Antitumor Effect
**Pancreatic Cancer**				
[23]	i.v.	5 × 10^7^ CFU	Polytherapy	BEV + GEM → A1-R > BEV + GEM > GEM > Ct
[24]	i.p.	1.5 × 10^8^ CFU	Monotherapy	A1-R > GEM or CDDP or 5FU > Ct
[36]	i.v.	5 × 10^7^ CFU	Monotherapy Polytherapy	A1-R > Ct A1-R + GEM > A1-R or GEM + nPTX or GEM or Ct
**Soft Tissue Sarcoma**				
(FDCS) [25]	i.p.	2 × 10^7^ CFU	Monotherapy Polytherapy	A1-R > Ct A1-R → DOX > Ct, A1-R → BEZ > Ct
(UPS) [27]	i.t.	5 × 10^7^ CFU	Monotherapy Polytherapy	A1-R > Ct A1-R → DOX > Ct
(Ewing’s sarcoma) [28]	i.v./i.t.	5 × 10^7^ CFU	Monotherapy Polytherapy	A1-R > Ct A1-R + DOX > Ct
(Pleomorphic liposarcoma) [33]	i.v.	5 × 10^7^ CFU	Monotherapy	A1-R > DOX or Ct
(USTS) [37]	i.v.	5 × 10^7^ CFU	Monotherapy	A1-R > DOX > Ct
(Myxofibrosarcoma) [39]	i.v.	5 × 10^7^ CFU	Monotherapy	A1-R > DOX or Ct
**Melanoma**				
[26]	i.v.	5 × 10^7^ CFU	Monotherapy Polytherapy	A1-R > Ct A1-R + TEM > A1-R, A1-R + TEM > TEM
[29]	i.v.	5 × 10^7^ CFU	Monotherapy Polytherapy	A1-R > Ct A1-R + TEM or A1-R + VEM > A1-R
[30]	i.v.	5 × 10^7^ CFU	Monotherapy Polytherapy	A1-R > Ct A1-R + VEM > COB + VEM or COB or VEM or A1-R or Ct
[34]	i.v.	5 × 10^7^ CFU	Monotherapy Polytherapy	A1-R > Ct A1-R + rMETase > TEM + rMETase or rMETase or TEM or Ct
**Osteosarcoma**				
[31]	i.v/i.a.	5 × 10^7^ CFU (i.v.) 5 × 10^5^ CFU (i.a.)	Monotherapy	A1-R (i.a.) > A1-R (i.v.) or CDDP or Ct, A1-R (i.v.) > Ct
[35]	i.v.	5 × 10^7^ CFU	Monotherapy Polytherapy	A1-R > CDDP or Ct A1-R + rMETase + CDDP > A1-R + rMETase > A1-R or rMETase or CDDP or Ct
**GIST**				
[32]	i.v.	5 × 10^7^ CFU	Monotherapy	A1-R > IMA or Ct
**Cancer of Unknown Primary**				
[38]	i.v.	5 × 10^7^ CFU	Monotherapy	A1-R > Ct

PDOX—patient-derived orthotopic xenograft; VEGF—vascular endothelial growth factor; i.v.—intravenous injection; CFU—colony-forming unit; BEV—bevacizumab; GEM—gemcitabine; A1-R—*S. typhimurium* A1-R; Ct—untreated control; i.p.—intraperitoneal injection; CDDP—cisplatinum; 5FU—fluorouracil; FDCS—follicular dendritic-cell sarcoma; DOX—doxorubicin; BEZ—dactolisib; TEM—temozolomide; UPS—undifferentiated pleomorphic sarcoma; i.t.—intratumoral injection; VEM—vemurafenib; COB—cobimetinib; i.a.—intra-arterial injection; GIST—gastrointestinal stromal tumor; IMA—imatinib; rMETase—recombinant methioninase; > or < indicates significant difference in treatment effect between groups; → indicates metachronous combination treatment protocol; + indicates synchronous combination treatment protocol.

## Dedication

This paper is dedicated to the memory of A. R. Moossa, M.D. and Sun Lee, M.D.
